# Bilateral Congenital Absence of Small Finger Flexor Digitorum Superficialis Tendons in a Trauma Patient

**DOI:** 10.7759/cureus.6948

**Published:** 2020-02-11

**Authors:** Jordan T Carter, Michael Polmear, Fernando Herrera, Gilberto Gonzalez

**Affiliations:** 1 Orthopaedics, Texas Tech University Health Sciences Center, El Paso, USA; 2 Plastic Surgery, Medical University of South Carolina, Charleston, USA

**Keywords:** flexor digitorum superficialis, congenital absence, anatomy, hand, physical examination

## Abstract

The flexor digitorum superficialis (FDS) is the only muscle in the intermediate layer of the flexor compartment of the forearm. Its main function is flexion of the proximal interphalangeal (IP) joint. Variations of the FDS are common, and knowledge of these variations is necessary for hand surgeons because the little finger tendon of the FDS is commonly used in hand reconstruction surgery. Here we present a case of bilateral absence of the little finger tendon of the FDS in an 11-year-old Hispanic female trauma patient presenting to the Emergency Department with multiple traumatic injuries including bilateral hand lacerations sustained in a motor vehicle accident. On physical examination, flexion of the IP joint of the thumb, and metacarpal phalangeal, proximal IP, and distal IP joints of the little finger were absent bilaterally. In the operating theater, the lacerations were extended to evaluate the status of the tendons of the FDS and flexor digitorum profundus (FDP). On the right, a complete transection of the FDP tendons to the ring and little fingers were found and repaired. Upon further exploration, the FDS tendon to the ring finger was identified and repaired, whereas the little finger tendon was found to be absent. On the left, the FPL tendon was identified and repaired along with the FDP tendon to the little finger. The FDS was subsequently identified and found to be lacking the tendon to the little finger. Clinically, the absence of the FDS could lead to problems in hand reconstruction surgery and functional testing of the hand.

## Introduction

The flexor digitorum superficialis (FDS) is the only muscle in the intermediate layer of the anterior forearm [1]. It has three heads originating from the medial epicondyle of the humerus, coronoid process of the proximal ulna, and anteroproximal radius [2]. The humeral and ulnar heads typically fuse to form the humeroulnar head [1-2]. Fibers from these three heads form a central belly in the proximal half of the forearm and then diverge into four beams [2]. The tendons of these beams insert into the radial and ulnar aspects of the proximal half of the middle phalanx at Camper’s chiasm of each digit except for the thumb [1]. The FDS is innervated by the median nerve and receives its blood supply from the ulnar artery.

 The primary action of FDS is to flex the middle phalanx at the proximal interphalangeal (PIP) joints and can also flex the metacarpal phalangeal (MCP) joints and wrist joint with sustained action [1,3]. This allows the human hand to make skilled, grasping movements necessary for fine motor skills [3-4]. For instance, Gupta and Kumar found that the independent function of the FDS tendons was essential for high caliber, professional musicians [3]. However, Austin et al. suggested that a common variant of a shared distal FDS tendon to the ring and small fingers limits independent PIP flexion of the small finger [5].

It is suggested that the flexors of the mammalian hand have evolved from the intrinsic muscles of the amphibian hand [4]. The origin of these muscles migrated proximally to establish the present separate forearm and intrinsic hand muscle arrangements, giving the organism more control over hand movements [4]. The evolutionary role of the FDS tendon to the small finger warrants further investigation, as its small structure has limited functional consequence [1,6] and its absence may represent evolutionary progression with improved independent flexion of the ring and small finger PIP joints [1,3,6].

 Anatomical variations of the forearm flexor muscles are common [2], and there are several documented variations of the FDS [1,3,5,7]. Variations in the FDS are clinically important for the assessment of injuries and pre-surgical planning for harvesting the small finger FDS tendon [3]. The purpose of this case report is to discuss a variant of congenital absence of bilateral small finger FDS tendons that was found on exploration of traumatic injuries.

## Case presentation

**Figure 1:**
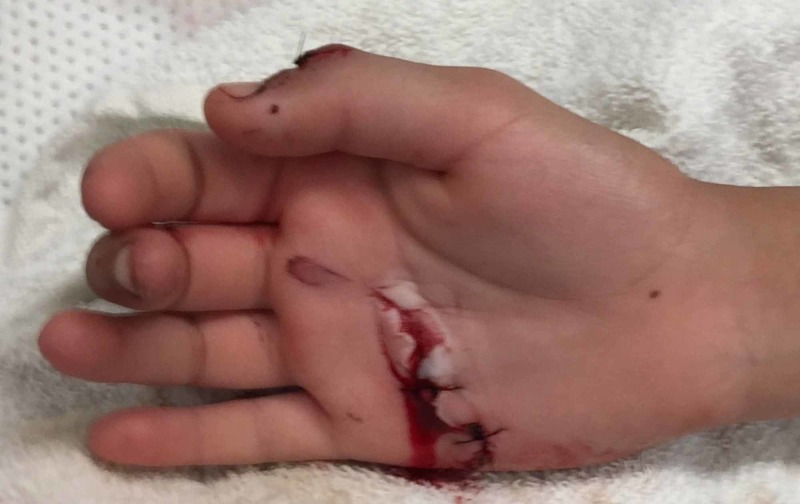
Preoperative view of injuries sustained to the right hand following in a motor vehicle accident.

An 11-year-old Hispanic female presented to the Emergency Department as a trauma patient with rib fractures, pelvic fracture, subarachnoid hemorrhage, and bilateral hand lacerations sustained from a motor vehicle accident. On examination of the hands, the right hand sustained a 1-cm dorsal transverse laceration just distal to the thumb interphalangeal (IP) joint and a second 3-cm volar transverse laceration overlying the ring and small finger metacarpal necks (Figure 1). There were no fractures identified in bilateral hands. On testing, there was an absence of thumb IP joint extension, and small and ring finger MCP, PIP, and distal IP (DIP) joints flexion, including isolated digital joint flexion testing with all other digits held in extension. Otherwise, flexion and extension of the MCP, PIP, and DIP joints of all digits were intact.

**Figure 2:**
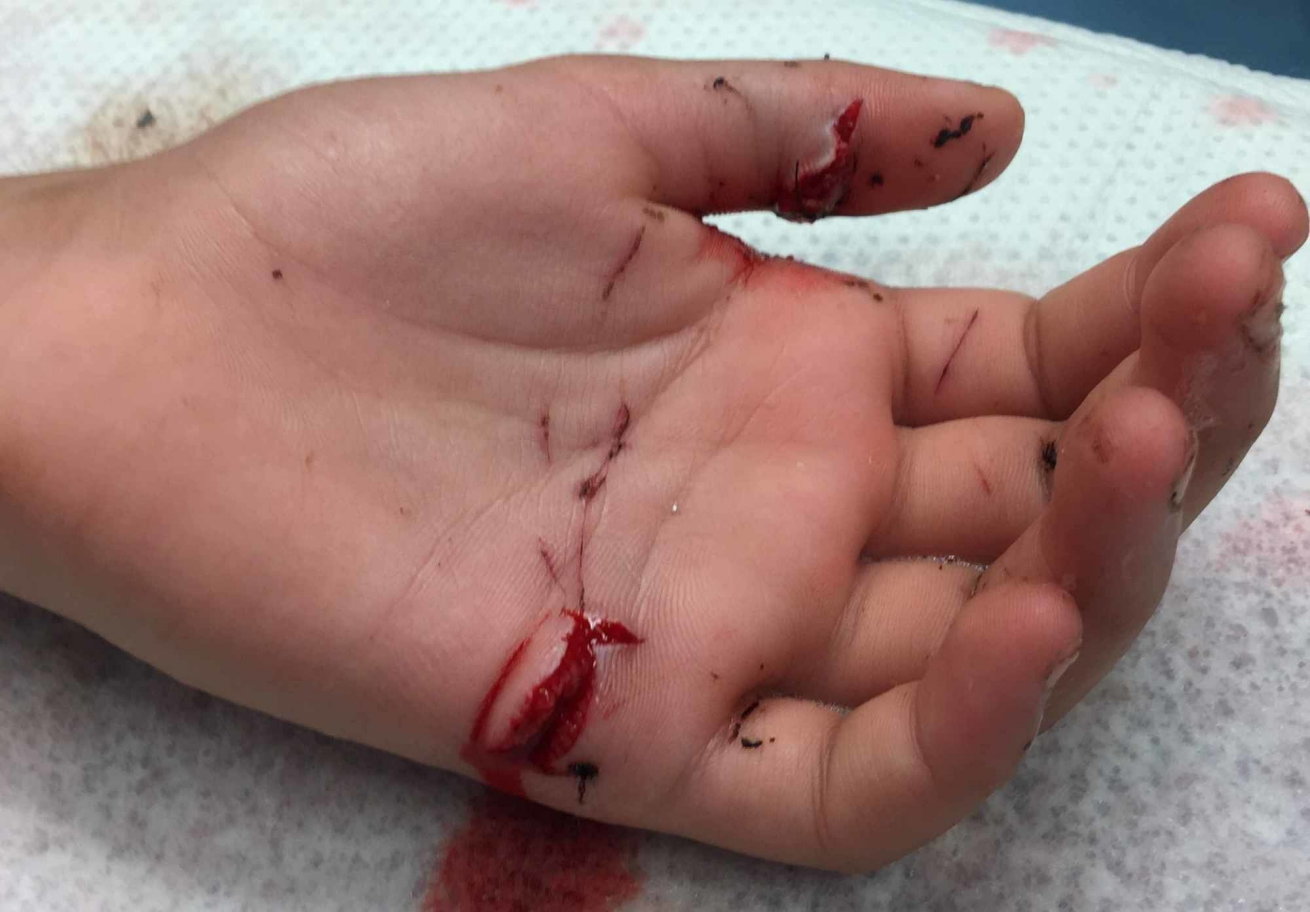
Preoperative view of injuries sustained to the left hand in a motor vehicle accident.

The left hand was found to have a 1-cm volar transverse laceration overlying the thumb (IP) joint and a second 1.5-cm volar transverse laceration overlying the small finger metacarpal neck (Figure 2). On testing, there was an absence of thumb IP joint flexion, and small finger MCP, PIP, and DIP joint flexion, including isolated digital joint flexion testing with all other digits held in extension. Otherwise, flexion and extension of the MCP, PIP, and DIP joints of all digits were intact.

**Figure 3:**
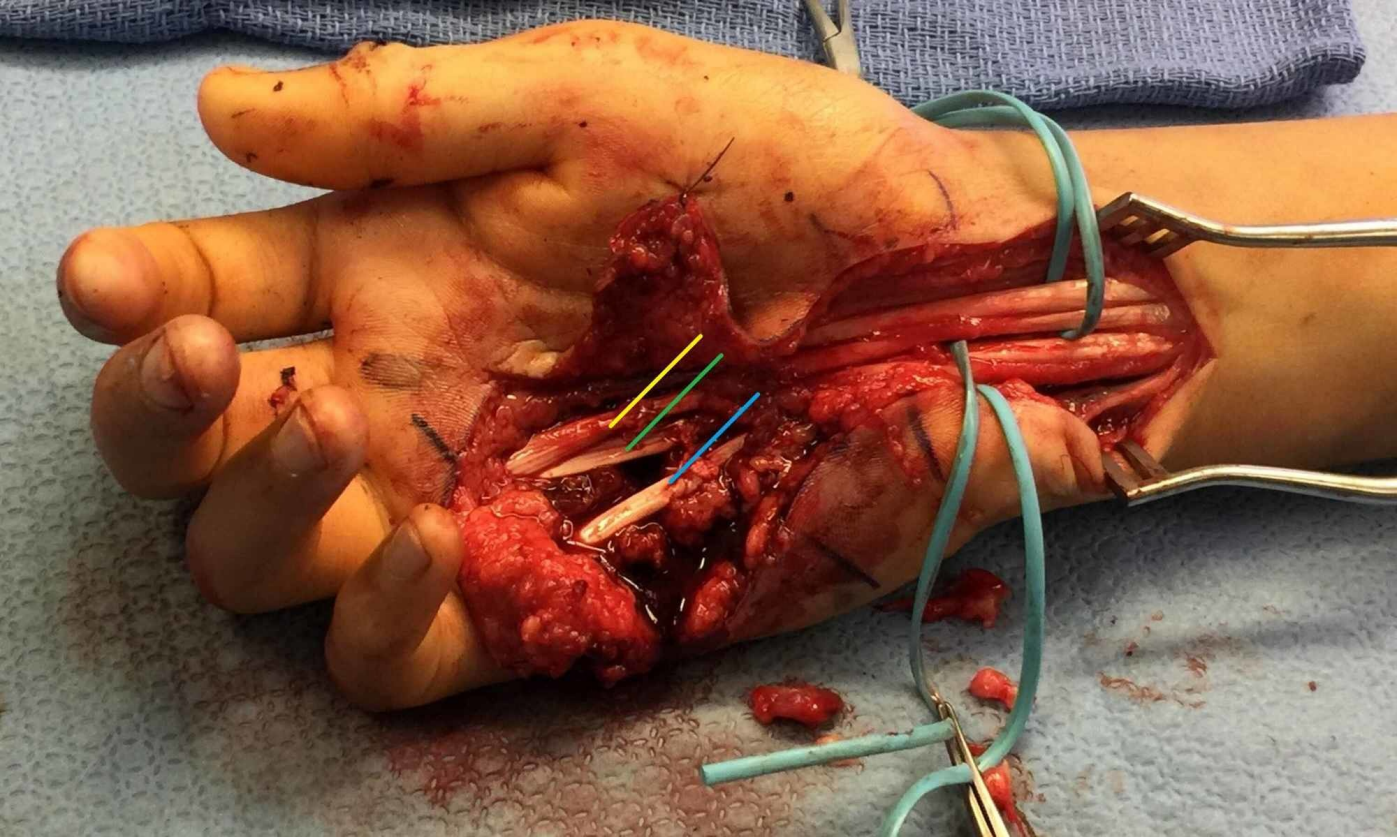
Intraoperative view showing the absence of the small finger tendon of the FDS in the right hand. Radial-sided vessel loop demarcates FDP tendons. Volar-sided vessel loop demarcates FDP tendons. There are two tendons noted on the ring finger (FDS and FDP); however, only one is present on the little finger (FDP). Yellow line indicates FDS tendon to the ring finger, green line indicates FDP tendon to the ring finger, and blue line indicates FDP tendon to the little finger. FDS, flexor digitorum superficialis; FDP, flexor digitorum superficialis

In the operating theater, the patient was placed supine on a hand table. Beginning with the right hand, the dorsal thumb laceration was extended and explored, and the extensor pollicis longus was repaired using 3/0 Supramid non-absorbable suture (S. Jackson Inc., Alexandria, VA). The volar laceration overlying the ring finger and small finger metacarpal necks was extended proximally through the transverse carpal ligament to identify retracted tendons. Upon exploration, the absence of the FDS tendon to the small finger was identified after identifying the tendon to the ring finger (Figure 3). Complete lacerations of the flexor digitorum profundus (FDP) tendons of the ring and small fingers and FDS tendon of the ring fingers were identified and repaired using 3/0 Supramid. Neurorrhaphy was performed on the common digital nerve to the fourth webspace and on the ulnar digital nerve to the small finger. The skin was closed primarily. 

**Figure 4:**
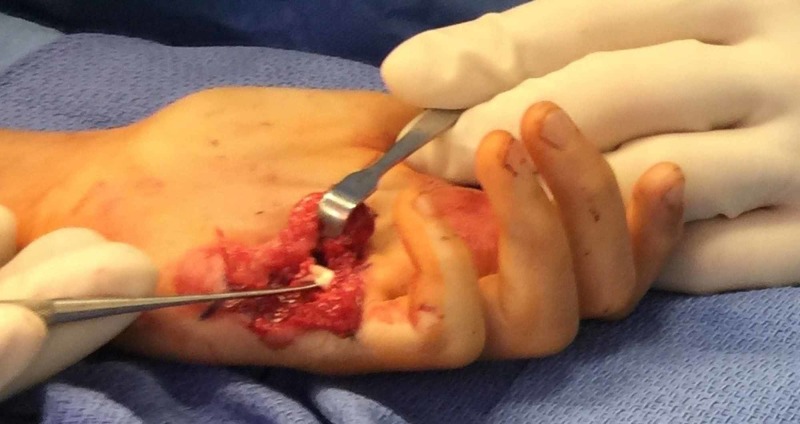
Intraoperative view showing the absence of the small finger tendon of the flexor digitorum superficialis in the left hand. Freer elevator is placed deep to repair the flexor digitorum profundus tendons of the small finger.

On the left side, the volar laceration around the thumb IP joint was extended, and the flexor pollicis longus tendon was repaired using 3/0 Supramid suture. Neurorrhaphy was performed on the radial and ulnar digital nerves of the thumb. The volar laceration overlying the small finger metacarpal neck was extended to identify the absence of the FDS tendon to the small finger and complete laceration of the FDP tendon to the small finger (Figure 4), which was repaired using 3/0 Supramid. Neurorrhaphy was performed on the ulnar digital nerve to the small finger. The skin was then closed primarily.

## Discussion

The bilateral absence of the FDS tendons of the small fingers in this patient was an incidental finding discovered during surgical exploration following bilateral hand trauma. The patient and parents denied any noticeable functional deficits before injury regarding the patient’s dexterity and were unaware of the bilateral absence of small finger FDS tendons. Recognition of this anatomic variant by the surgeon improved surgical exploration and treatment of this patient.

With a reported incidence between 0.25% in the Indian population to as high as 21% in the Caucasian population, the congenital absence of the FDS is not an uncommon finding and seems to vary depending on ethnicity [1]. However, to our knowledge, there are currently no published studies on the epidemiology of congenital FDS absence in the Hispanic population.

An important clinical assumption in functional testing of the hand is symmetrical function [5]. However, in this patient, both sides would have been similar due to the bilateral absence. Physical examination maneuvers for isolating function of the FDS from the FDP, particularly within the small finger, are often confounded by FDS variants. Recent studies have described the diagnostic accuracy of physical examination maneuvers for FDS function and variants. One maneuver is the Expanded Baker Modified Exam, where all fingers are held in extension by the examiner except the digit of interest, and then the examinee is asked to flex the PIP and DIP joints independently to test the FDS and the FDP, respectively [8]. This examination is limited in the diagnosis of FDS variants, as flexion is a binary outcome and only suggests whether FDS and FDP are intact [9]. A new method proposed by Tan et al. aims to diagnose variants in the ring and small finger FDS and FDP tendons [9]. In this method, instead of forcefully holding the fingers in extension, possibly creating a diversionary effect, the hands are held with the palms in contact facing each other with corresponding MCP, PIP, and DIP joints in extension. The examinee is asked to flex the PIP joint of the small fingers. If the patient can touch the dorsal aspect of the contralateral hand without flexing the DIP joints, then the FDS tendon function is independent. A connection between the ring and small finger FDS tendons was further graded as loose or close by relative flexion of the PIP joint of the adjacent digit. FDP substitution or FDS deficiency of the small finger was diagnosed based on the inability to flex the PIP joint independent of DIP joint flexion [9].

There are many described variations of the FDS [1,3,5,7-10], and suspicion of these variations preoperatively and identification intraoperatively is important for accurate diagnosis and tendon repair. The FDS independent variation, where the small finger FDS tendon inserts on to the middle phalanx of the small finger independently without direct connections to the ring finger tendon, is the most common variant of the FDS [2,5,8,9]. However, abnormalities, such as the isolated unilateral absence of the FDS [7], unilateral absence of the tendon of the FDS to the little finger [2,5], conjoint ring and small finger digit tendons of the FDS [9], unilateral absence of the FDS and palmaris longus muscle [1,6,10], isolated bilateral absence of the FDS little finger tendons [3], and bilateral absence of the FDS in association with and without absence of the palmaris longus [1,6,7,10], have been described in the literature, with the majority of the knowledge about these variants coming from cadaveric studies. One surgical implication of the variant seen in this patient (the bilateral absence of the little finger tendon of the FDS variant) is that the small finger tendon of the FDS is not available for autograft in hand reconstruction surgery [3].

## Conclusions

The finding of bilateral absence of small finger FDS tendons in this patient describes a novel variant of the FDS in a living subject, as well as potential implications of FDS variants on the physical examination. Knowledge of anatomic FDS variants may improve intraoperative diagnosis and minimize additional exploration.
